# Nonintubated spontaneous ventilation versus intubated mechanical ventilation anesthesia for video-assisted thoracic surgery in terms of perioperative complications and practitioners’ workload assessments: a pilot randomized control study

**DOI:** 10.1186/s12871-024-02481-1

**Published:** 2024-03-12

**Authors:** Xian-gang Kong, Kun Wang, Yu-tao Wei, Bo Sun, Guo-dong Gao, Cheng-wei Song, Cheng-wen Li

**Affiliations:** 1 Department of Anesthesiology, Jining No. 1 People’s Hospital, No. 6 Jiankang Road, Rencheng District, Jining, 272011 China; 2 Department of Thoracic Surgery, Jining No. 1 People’s Hospital, No. 6 Jiankang Road, Rencheng District, Jining, 272011 China; 3 Department of Anesthesiology, Beijing Friendship Hospital, Capital Medical University, No. 95 Yongan Road, Xicheng District, Beijing, 100050 China

**Keywords:** Spontaneous ventilation, Nonintubated, Mechanical ventilation, Intubation, Video-assisted thoracoscopic surgery

## Abstract

**Background:**

The use of nonintubated video-assisted thoracoscopic surgery (NI-VATS) has been increasingly reported to yield favourable outcomes. However, this technology has not been routinely used because its advantages and safety have not been fully confirmed. The aim of this study was to assess the safety and feasibility of nonintubated spontaneous ventilation (NI-SV) anesthesia compared to intubated mechanical ventilation (I-MV) anesthesia in VATS by evaluating of perioperative complications and practitioners’ workloads.

**Methods:**

Patients who underwent uniportal VATS were randomly assigned at a 1:1 ratio to receive NI-SV or I-MV anesthesia. The primary outcome was the occurrence of intraoperative airway intervention events, including transient MV, conversion to intubation and repositioning of the double-lumen tube. The secondary outcomes included perioperative complications and modified National Aeronautics and Space Administration Task Load Index (NASA-TLX) scores from anesthesiologists and surgeons.

**Results:**

Thirty-five patients in each group were enrolled in the intention-to-treat analysis. The incidence of intraoperative airway intervention events was greater in the NI-SV group than in the I-MV group (12 [34.3%] vs. 3 [8.6%]; OR = 0.180; 95% CI = 0.045–0.710; *p* = 0.009). No significant difference was found in the postoperative pulmonary complications between the groups (*p* > 0.05). The median of the anesthesiologists’ overall NASA-TLX score was 37.5 (29–52) when administering the NI-SV, which was greater than the 25 (19-34.5) when the I-MV was administered (*p* < 0.001). The surgeons’ overall NASA-TLX score was comparable between the two ventilation strategies (28 [21-38.5] vs. 27 [20.5–38.5], *p* = 0.814).

**Conclusion:**

The NI-SV anesthesia was feasible for VATS in the selected patients, with a greater incidence of intraoperative airway intervention events than I-MV anesthesia, and with more surgical effort required by anesthesiologists.

**Trial registration:**

Chinese Clinical Trial Registry, ChiCTR2200055427. https://www.chictr.org.cn/showproj.html?proj=147872 was registered on January 09, 2022.

**Supplementary Information:**

The online version contains supplementary material available at 10.1186/s12871-024-02481-1.

## Introduction

Video-assisted thoracoscopic surgery (VATS) has been developed and widely used due to minor trauma and rapid recovery [1]. The uniportal approach is a more minimally invasive form of VATS [2]. Conventional VATS is commonly performed under double-lumen intubated anesthesia with one-lung ventilation as the standard technique. This technique can fully expose the surgical field and perfectly isolate the operative lung [3], and may also leads to intubation- and mechanical ventilation (MV)-induced injuries [4–6].

Nonintubated spontaneous ventilation (NI-SV) anesthesia with a laryngeal mask (LMA) may be a suitable alternative to conventional intubated mechanical ventilation (I-MV) anesthesia with a double-lumen tube (DLT) for VATS in selected patients. Evidence has demonstrated the potential advantages of nonintubated VATS, such as reduced intubation- and MV-related complications, avoidance of postoperative muscle paralysis due to muscle relaxant residues, decreased opioid-related side effects, and enhanced recovery after surgery [7, 8]. However, the advantages and safety of this nonintubated strategy have not been fully confirmed [9–12]. Thus, at present, the NI-SV strategy has not yet been routinely adopted for VATS, mainly owing to the potential risk of an unsafe airway and the concerns of poor surgical conditions, which emphasize the need for anesthesiologists to be vigilant of the surgical process and to act accordingly for providing the adequate anesthetic conditions.

Therefore, we designed a randomized controlled trial (RCT) to evaluate the safety and feasibility of NI-SV anesthesia with an LMA compared to I-MV anesthesia with a DLT in uniportal VATS, with a focus on perioperative complications and practitioners’ workload evaluation for both scenarios. Based on the hypothesis that adequate SV and adequate intraoperative anesthetic conditions are fraught with difficulties when administering NI-SV anesthesia for VATS, the occurrence of intraoperative airway intervention events was investigated for both intervention strategies, including conversion to mechanical ventilation or intubation under NI-SV anesthesia and repositioning of the DLT and double-lung ventilation under I-MV anesthesia.

## Materials and methods

### Ethics and registration

This RCT was approved by the ethical committee of Jining No. 1 People’s Hospital (No. 2021[0027]) and was performed in accordance with the Declaration of Helsinki. The study protocol was registered in the Chinese Clinical Trials Registry (ChiCTR2200055427, 09/01/2022) and followed the consolidated Standards of Reporting Trials (CONSORT) guidelines. Written informed consent was obtained from all patients before randomization.

### Participants

Seventy patients who underwent elective uniportal VATS at Jining No. 1 People’s Hospital with American Society of Anesthesiologists (ASA) physical status grade I-II and Mallampati class I-II were enrolled in this study. The exclusion criteria were as follows: previous history of thoracic surgery; preoperative examination revealing extensive pleural adhesion; predictable difficult airway; body mass index > 30 kg/m^2^; high risk of regurgitation; comorbidity with severe heart, lung or brain diseases; coagulation dysfunction; and refusal to participate in the study. Both anesthesiologists in charge of anesthesia and surgeons in charge of surgical procedures in this RCT trial had more than 5-years of experience in VATS.

### Randomization and blinding

Eligible patients were randomly assigned to receive NI-SV or I-MV anesthesia at a 1:1 ratio via computer-generated random numbers. The randomization results were sealed in sequentially numbered opaque envelopes and disclosed prior to anesthesia. It was easy to recognize the grouping of patients during the operation; thus, the surgeons and anesthesiologists performing the operation were not blinded to the group assignment.

### Anesthesia protocol

All patients accepted routine diet prohibition. Before anesthesia, visual laryngoscope, fiberoptic bronchoscopy, stethoscopy, LMA, and DLT instruments were prepared for tracheal intubation at any time. Upon patient arrival in the operating room, the electrocardiogram, invasive blood pressure, pulse oxygen saturation (SpO_2_), end-tidal carbon dioxide pressure (PetCO_2_) and bispectral index (BIS) were monitored continuously. After establishing upper limb vein access, crystalloid fluid was administered at 4–6 mL/(kg•h). Then, 100% oxygen at 5 L/min was delivered through a face mask.

Patients in both groups received ultrasound-guided thoracic paravertebral block (TPVB) before anesthesia induction. Preemptive intravenous infusion of 1.0 ug/(kg•h) dexmedetomidine for 10 min was given for the alleviation of nervousness before performing TPVB. Subsequently, the patients were placed in the lateral decubitus position. After positioning the T_5_ spinous process, the area where TPVB was performed was subjected to skin antisepsis and covered with aseptic hole-towels. A 5–8 MHz convex probe wrapped with a sterile sheath was placed parallel to the intercostal space on the level of the T_5_ transverse process, and paramedian oblique axial scanning was performed. When the transverse process, pleura, superior costotransverse ligament and thoracic paravertebral space were clearly visualized, a nerve block needle (21-gauge, 100 mm) was introduced 2–3 cm lateral to the probe and advanced in the caudal-cranial direction via the in-plane technique. Once the needle was advanced to reach the TPVS, 15 ml of 0.375% ropivacaine was administered, and pleural depression was observed.

In patients receiving NI-SV, anesthesia was induced with intravenous propofol (1.0–2.0 mg/kg) and sufentanil (0.2–0.4 ug/kg). When the BIS value decreased to less than 60, a suitable LMA was inserted and connected to the anesthesia machine. Anesthesia was maintained with propofol 2–6 mg/(kg•h), remifentanil 0.03–0.08 µg/(kg•min) and dexmedetomidine 0.5 µg/(kg•h) by intravenous infusion. Spontaneous ventilation was maintained during anesthesia and 100% oxygen was supplied via the LMA at a fresh gas flow of 4–5 L/min. After thoracoscopic incision, pulmonary surface and vagus nerve blockades were performed by the surgeons under direct thoracoscopic vision, in which 10 ml of 2% lidocaine was sprayed on the surface of the affected lung and 5 ml of 1% lidocaine was administered near the vagus nerve (right side in the upper mediastinum; left side in the aortopulmonary window) [13]. Sufentanil (0.1–0.2 ug/kg) was administered to alleviate the cough reflex or mediastinal flutter as necessary. In the case of hypoxemia (SpO_2_ < 92%) or hypercapnia (PetCO_2_ > 60 mmHg) during the operation, our first action was to supply positive pressure ventilation followed by simultaneous intermittent mandatory ventilation (SIMV) through the LMA to inflate the bilateral lung, and our last resort was to perform intubation in the lateral decubitus position. Converting to intubation was also considered in the event of severe intraoperative cough, mediastinal flutter or unsatisfactory lung collapse, which affects surgical manipulations, or persistent intraoperative hypoxemia, which requires continuous manual ventilation or SIMV. In these circumstances, a single-lumen tube combined with a bronchial blocker or a DLT should be placed under the guidance of a fiberoptic bronchoscope in the lateral decubitus position.

In patients receiving I-MV, anesthesia was induced with intravenous propofol (1.0–2.0 mg/kg), sufentanil (0.2–0.4 µg/kg) and cisatracurium (0.2–0.3 mg/kg). When the BIS value decreased to less than 60, a suitable DLT was inserted, and the proper tube position was confirmed via a fiberoptic bronchoscope. Subsequently, patients underwent two-lung ventilation with an intermittent positive pressure ventilation (IPPV) model (TV, 6–8 mL/kg; a fresh gas flow of 100% oxygen, 4–5 L/min; RR, 12–16 times/min; PEEP, 5 cm H_2_O; peak pressure, < 30 cm H_2_O). Unaffected-side one-lung ventilation with the IPPV model (TV, 4–5 mL/kg; a fresh gas flow of 100% oxygen, 4–5 L/min; RR, 12–16 times/min; PEEP, 5 cm H_2_O; peak pressure, < 30 cm H_2_O) was utilized for surgical procedures. Anesthesia was maintained with 2–6 mg/(kg•h) propofol, 0.03–0.08 µg/(kg•min) remifentanil, 0.5 µg/(kg•h) dexmedetomidine by intravenous infusion. Cisatracurium 0.05 mg/kg and sufentanil (0.1–0.2 µg/kg) were intravenously added according to the intraoperative conditions. Hypoxemia was corrected by adjusting the repositioning of the DLT, adjusting respiratory parameters and/or converting the DLT to double-lung ventilation. Neostigmine 0.04 mg/kg and atropine 0.02 mg/kg were given at the end of the skin suture.

In both groups, a BIS device was used to monitor the depth of anesthesia and the BIS value was maintained at 45–60 during anesthetic maintenance by adjusting the infusion rate of propofol and remifentanil. Cisatracurium was ceased 30 min before the predicted end of surgery. Dexmedetomidine was ceased when the thoracic cavity was closed, and propofol and remifentanil were ceased when the skin was sutured. Patients were transferred to the postanesthetic care unit (PACU) following the extubation of the LMA or DLT in the operating theater and then to the ward after they were fully awake.

All participants received patient-controlled intravenous analgesia (PCIA) with 2 µg/kg sufentanil and 5 mg tropisetron in a total volume of 100 ml (the parameters were a background dose of 2 ml/h, a bolus dose of 2 ml, and a lockout interval of 15 min) for 48 h postoperatively. If the visual analogue scale score > 4 and no pain relief was achieved by pressing the PCIA, 5 mg morphine was administered intravenously as a rescue analgesic.

### Surgical technique

In both groups, uniportal VATS was performed in the lateral position, with an incision ≤ 2 cm made in the fourth or fifth intercostal space on the anterior axillary line as the operation hole. Surgical procedures such as pulmonary wedge resection, segmentectomy or lobectomy were performed according to the location and nature of the tumour during the operation. Before the chest was closed, warm sterile saline solution was used to irrigate the thoracic cavity and the operated lung was reinflated by manual ventilation at a peak pressure less than 30 cm H_2_O via the LMA or DLT to test for air leakage. After placing a 16 Fr chest tube, the pleura, muscles, and skin were sutured in turn. Patients recovered to the supine position after the operation, and a H_2_O peak pressure less than 30 cm H_2_O was used to expand the lung again. The chest tube was removed when no air leakage was observed for 6 h and when the volume of thoracic drainage did not exceed 200 ml for 24 h.

### Outcome variables

#### Primary outcome variables

The primary outcome was the occurrence of intraoperative airway intervention events, which occurred intraoperatively to improve oxygenation or surgical conditions, including intraoperative changes in ventilation modes, such as conversion to MV or double-lung ventilation, and intraoperative replacement or repositioning of airway tools, such as conversion to intubation and repositioning of the DLT.

#### Secondary outcome variables

The secondary outcomes included perioperative complications and practitioner workload scores. The perioperative complications included intraoperative adverse events (hypoxemia, hypercapnia, cough reflex, mediastinal flutter, conversion to open thoracotomy, etc.), intensive care unit (ICU) admission, intubation-related injuries (lip injury, sore throat, hoarseness, etc.), and postoperative pulmonary complications ([PPCs] pneumonia, atelectasis, air leakage, pleural effusion, dyspnea, fever, etc.). Hypoxemia is defined as SpO_2_ < 92% for at least 1 min (i.e., two consecutive SpO_2_ recordings < 92% at a 1 min interval) during the interval between the start of opening the chest and the end of closing the chest [9, 10, 14]. Hypercapnia with an arterial carbon dioxide tension (PaCO_2_) < 75 mm Hg is not suggested as an indication of conversion to intubation and can be treated with low PEEP and pressure support on the circle [9]. Nonetheless, a PetCO_2_ value of 60 mmHg was the permissive upper limit in this study for safety purposes due to the inconsistency between PetCO_2_ and PaCO_2_ during one-lung ventilation [15].

The practitioners’ workload was assessed immediately at the end of the operation using the modified National Aeronautics and Space Administration Task Load Index (NASA-TLX) scores by anesthesiologists in charge of anesthesia and surgeons in charge of surgical procedures. The Chinese edition of the NASA-TLX, with a modification of removing the self-performance dimension, is a validated measure of workload with a Cronbach’s α of 0.645 [16], comprising five predefined dimensions: mental demand, physical demand, temporal demand, effort, and frustration levels. Each of the dimensions is weighted on a 100-point scale (0 = very low, 100 = very high) in increments of 5 points by the subject during the application of the instrument, and scores are presented as the dimension score and the average overall score. A previous study of surgeons from different specialties also demonstrated a poor association between reported mental and physical demands and self-performance, especially when there was deviation from the expected procedural difficulty [17]. The greater the NASA-TLX score was, the greater the workload involved in executing the task [18].

Surgical field exposure was also scored at the end of the operation by surgeons in charge of surgical procedures on a 4-point scale (1 = Satisfactory; 2 = Clear, lung collapse not good, but does not need to interrupt the operation; 3 = Poor, lung collapse not satisfactory, need to interrupt the operation; 4 = Very poor, surgery cannot be completed, need to convert to intubation). Intraoperative anesthetic requirements (propofol, remifentanil, and sufentanil), extubation time, length of stay in the PACU and postoperative admission to the intensive care unit (ICU) were also recorded.

### Sample size calculation

The sample size was estimated based on the incidence of intraoperative airway intervention events. In our pilot study with 10 patients in each group, intraoperative airway intervention events occurred in 4 patients (40%) who received NI-SV anesthesia and 1 patient (10%) who received I-MV anesthesia. A sample size of 26 participants in each group was calculated via PASS 15.0, with a type I error of 0.05, a power of 80% and an allocation ratio of 1.0. Considering the dropout rate, a final sample size of 70 patients was recruited for the study.

### Statistical analysis

All the data were analyzed by SPSS version 26.0 (IBM, Armonk, NY, USA). The occurrences of intraoperative airway intervention events and perioperative complications were expressed as numbers (percentages) and were analyzed using the chi-square test or the Fisher’s exact test in both the intention-to-treat and per-protocol populations at odds ratios (ORs) and 95% confidence intervals (CIs). All continuous data are presented as median and interquartile range and were compared using the Mann–Whitney *U* test. *P* < 0.05 was considered to indicate statistical significance.

## Results

### Patient characteristics

**Fig. 1:**
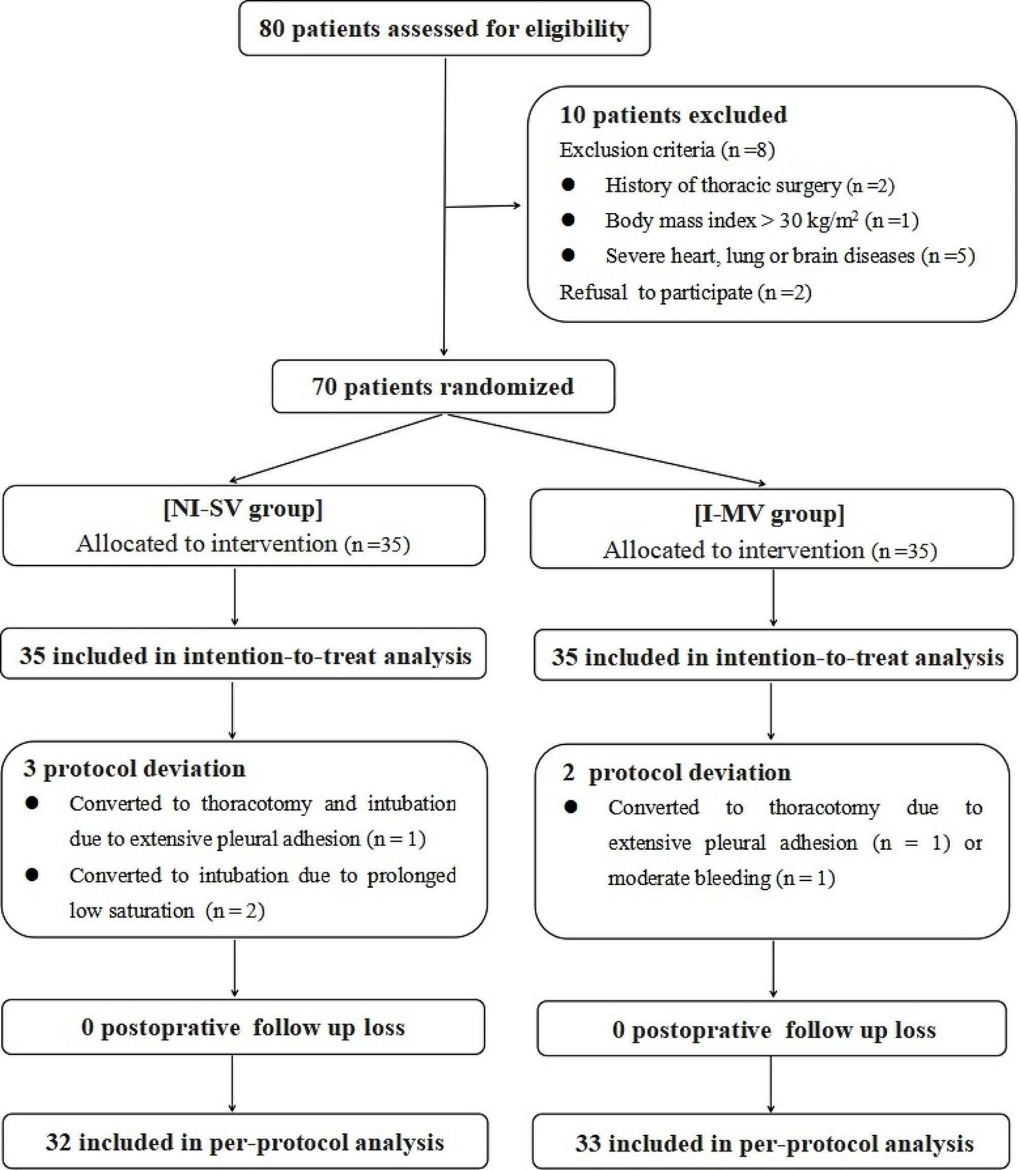
Consolidated standards of reporting trials flow diagram. NI-SV: non-intubated spontaneous ventilation; I-MV, intubated mechanical ventilation

From January 2022 to May 2022, a total of 80 patients were assessed for eligibility in this study, 10 of whom were excluded because they did not meet the inclusion criteria (*n* = 8) or refused to participate (*n* = 2). The remaining 70 patients were randomized into 2 groups (35 patients in each group), and all were enrolled in the analysis of the intention-to-treat population. After 5 protocol deviations (2 I-MV patients who underwent conversion to open thoracotomy, and 3 NI-SV patients who underwent conversion to intubation or open thoracotomy), 65 patients were enrolled in the analysis of the per-protocol population (Fig. 1). There were no significant differences in demographic data between the two groups (*p* > 0.05), whether in the intention-to-treat population or in the per-protocol population (Table 1).

**Table 1:** Patient demographics

	Intention-to-Treat		*p* value	Per-Protocol		*p* value
	NI-SV (*n* = 35)	MV (*n* = 35)		NI-SV (*n* = 32)	MV (*n* = 33)	
Age (year)	58(47.5–65)	58(52.5–65)	0.810	58.5(47.5–66.5)	58(52–65)	0.963
Gender			0.811			0.890
Male	16(45.7)	17(48.6)		14(43.8)	15(45.5)	
Female	19(54.3)	18(51.4)		18(56.3)	18(54.5)	
Height (cm)	166(160-171.5)	166(160–170)	0.799	165.5(160-171.5)	166(160–170)	0.827
Weight (kg)	65(59.5–74)	63(60-70.5)	0.680	65(59.5–73)	63(60–70)	0.540
BMI (kg/m^2^)	23.9(22.2–26.1)	23.5(21.8–26.3)	0.833	23.7(22.2–25.8)	23.4(21.3–25.1)	0.600
ASA grade			0.806			0.724
I	14(40.0)	13(37.1)		13(40.6)	12(36.4)	
II	21(60.0)	22(62.9)		19(59.4)	21(63.6)	
Comorbidities						
Diabetes mellitus	5(14.3)	3(8.6)	0.710	4(12.5)	3(9.1)	0.482
Hypertension	6(17.1)	9(25.7)	0.382	6(18.8)	8(24.2)	0.590
Preoperative lung function						
FVC (L)	3.54(2.90–4.27)	3.27(2.88–3.89)	0.428	3.45(2.90–4.20)	3.27(2.85–3.88)	0.447
FEV1 (L)	2.66(2.26–3.27)	2.88(2.47–3.15)	0.456	2.65(2.21–3.35)	2.87(2.48–3.12)	0.524
FEV1/FVC (%)	79.6(73.2–82.3)	80.2(76.6–84.0)	0.310	79.5(73.2–82.3)	80.2(77.3–83.3)	0.319

Data are presented as medians (interquartile ranges) or numbers (percentages) when appropriate

NI-SV: nonintubated spontaneous ventilation; I-MV: intubated mechanical ventilation; BMI: body mass index; ASA: American Society of Anesthesiologists; FVC: forced vital capacity; FEV1, forced expiratory volume in the first second

### Intraoperative airway intervention events

The occurrence of intraoperative airway intervention events was significantly greater in the NI-SV group than in the I-MV group, both in the intention-to-treat population (34.3% vs. 8.6%; OR = 0.180; 95% CI = 0.045–0.710; *p* = 0.009) and in the per-protocol population (31.3% vs. 9.1%; OR = 0.220; 95% CI = 0.054–0.894; *p* = 0.026), as shown in Table 2.

**Table 2:** Intraoperative airway intervention events

	Intention-to-Treat		OR (95% CI)	*p* value	Per-Protocol		OR (95% CI)	*p* value
	NI-SV (*n* = 35)	MV (*n* = 35)			NI-SV (*n* = 32)	MV (*n* = 33)		
Intraoperative airway intervention events	12(34.3)	3(8.6)	0.180(0.045–0.710)	0.009	10(31.3)	3(9.1)	0.220(0.054–0.894)	0.026
Transient mechnanical ventilation	10(31.3)	--		Na	10(31.3)	--		Na
Conversion to intubation	3(8.6)	--		Na	0(0)	--		Na
Repositioning of the DLT	--	2(5.7)		Na	--	2(6.1)		Na
Conversion to double-lung ventilation	--	1(2.9)		Na	--	1(3.0)		Na

Data are presented as numbers (percentages) or ORs (95% CIs) when appropriate

NI-SV: nonintubated spontaneous ventilation; I-MV: intubated mechanical ventilation; OR: odds ratio; CI: confidence interval; DLT: double-lumen tube; Na: not applicable

### Perioperative complication

In both the intention-to-treat and per-protocol populations, the intraoperative complications of hypoxemia (SpO_2_ < 92%), hypercapnia (PetCO_2_ > 60 mm Hg) and cough reflex occurred more frequently in the NI-SV patients than in the I-MV patients (*p* < 0.05). Although the intubaion-related complications of sore throat and hoarseness were more prevalent in the I-MV patients than in the NI-SV patients in the per-protocol analysis (*p* < 0.05), no significant difference in these complications was observed between the two groups in the intention-to-treat analysis (*p* > 0.05). No significant difference was found in terms of PPCs between the two groups (*p* > 0.05), as shown in Table 3.

**Table 3:** Perioperative complications

	Intention-to-Treat		*p* value	Per-Protocol		*P* value
	NI-SV (*n* = 35)	MV (*n* = 35)		NI-SV (*n* = 32)	MV (*n* = 33)	
Intraoperative complications						
Hypoxemia	12(34.3)	3(8.6)	0.009	10(31.3)	3(9.1)	0.026
Hypercapnia ^†^	11(31.4)	0(0)	< 0.001	10(31.3)	0(0)	< 0.001
Mediastinal flutter	0 (0)	0(0)	1.000	0 (0)	0(0)	1.000
Cough reflex	9(25.7)	1(2.9)	0.006	7(21.9)	1(3.0)	0.027
Conversion to open thoracotomy	1(2.9)	2(5.7)	1.000	0(0)	0(0)	1.000
Intubation-related complications						
Lip injury	1(2.9)	2(5.7)	1.000	1(3.1)	2(6.1)	1.000
Sore throat	7(20.0)	14(40.0)	0.068	4(12.5)	12(36.4)	0.026
Hoarseness	2(5.7)	7(20.0)	0.151	0(0)	6(18.2)	0.024
PPCs						
Pneumonia	3(8.6)	4(11.4)	1.000	1(3.1)	3(9.1)	0.613
Atelectasis	3(8.6)	4(11.4)	1.000	2 (6.3)	4(12.1)	0.672
Air leakage at 24 h	2(5.7)	4(11.4)	0.673	1(3.1)	4(12.1)	0.355
Pleural effusion	1(2.9)	1(2.9)	1.000	0(0)	1(3.0)	1.000
Fever	1(2.9)	2(5.7)	1.000	1(3.1)	2(6.1)	1.000
Dyspnea	2(5.7)	6(17.1)	0.259	1(3.1)	5(15.2)	0.197

Data are presented as numbers (percentages)

NI-SV: nonintubated spontaneous ventilation; I-MV: intubated mechanical ventilation; PPCs: postoperative pulmonary complications

^†^ PetCO_2_ > 60 mm Hg

### Practitioners’ workload scores

Both the analyses of the intention-to-treat and per-protocol populations showed that the anesthesiologists’ overall NASA-TLX score and the dimensional scores of mental demand, temporal demand, effort, and frustration level were significantly greater when administering NI-SV anesthesia than when I-MV anesthesia was done (*p* < 0.05). In both the analyses of the intention-to-treat and per-protocol populations, there were no significant differences in the surgeons’ overall NASA-TLX score or each dimensional score between patients who underwent surgical procedure under NI-SV anesthesia and those who underwent surgical procedures under I-MV anesthesia (*p* > 0.05), as shown in Table 4.

**Table 4:** Modified NASA-TLX scores

Workload	Intention-to-Treat		*p* value	Per-Protocol		*p* value
	NI-SV (*n* = 35)	MV (*n* = 35)		NI-SV (*n* = 32)	MV (*n* = 33)	
Assessed by anesthesiologists						
Mental demand	45(40–55)	35(25–45)	0.021	45(37.5–55)	35(25–45)	0.040
Physical demand	40(30–50)	30(25-42.5)	0.092	35(27.5–50)	30(25–45)	0.232
Temporal demand	45(35–55)	20(10–30)	< 0.001	45(32.5–55)	20(10–30)	< 0.001
Effort	40(30–55)	30(15–35)	< 0.001	40(30–55)	30(20–35)	0.001
Frustration level	25(10–45)	10(10-22.5)	0.008	20(10–45)	15(10–25)	0.034
The overall score	37(29.5–52)	25(19-34.5)	< 0.001	37.5(29–52)	25(19–35)	0.001
Assessed by surgeons						
Mental demand	40(25–45)	35(25–45)	0.901	30(25–45)	35(25–45)	0.868
Physical demand	30(22.5–45)	35(22.5–45)	0.745	32.5(22.5–45)	35(25–45)	0.905
Temporal demand	20(10-27.5)	20(10–30)	0.986	17.5(10–30)	20(10–30)	0.979
Effort	35(25–45)	30(25–45)	0.561	35(25–45)	30(25–45)	0.556
Frustration level	15(10–30)	20(10–30)	0.967	17.5(10–30)	20(10–30)	0.931
The overall score	28(21-38.5)	27(20.5–38.5)	0.814	28(19–39)	27(21–38)	0.753

Data are presented as medians (interquartile ranges)

NI-SV: nonintubated spontaneous ventilation; I-MV: intubated mechanical ventilation; NASA-TLX: National Aeronautics and Space Administration Task Load Index

### Patient surgical and anesthetic characteristics

No significant differences were observed with respect to the durations of anesthesia and surgery, surgery type, surgical field exposure score, requirements for propofol and remifentanil, or postoperative ICU admission between the NI-SV and I-MV groups in either the analyses of the intention-to-treat or per-protocol populations (*p* > 0.05). Compared with those in the I-MV group, the requirments for sufentanil (mainly for anesthetic induction), minimum intraoperatove SpO_2_, extubation time and PACU stay were significantly lower (*p* < 0.05), while the maximum intraoperatove PetCO_2_ was greater in the NI-SV group (*p* < 0.05), both in the intention-to-treat population and in the per-protocol population (Supplementary Table 1).

## Discussion

The results of this study based on the analysis of the intention-to-treat population showed a comparable incidence of intubation-related injuries and PPCs between the NI-SV and I-MV strategies but a greater incidence of intraoperative airway intervention events in NI-SV patients. Moreover, the NASA-TLX results from both the analyses of the intention-to-treat and per-protocol populations indicated that the anesthesiologists in charge experienced a greater workload during the NI-SV, while the surgeons in charge experienced an equal workload across the two ventilation strategies.

Sedation is not mandatory but is preferred for reducing accidental body movement and discomfort during NI-SV VATS. However, sedation-induced respiratory depression and the iatrogenic pneumothorax following thoracotomy have a synergetic role in impairing lung function. Conversion to I-MV is suggested for persistent hypoxemia (PaO_2_ < 60 mmHg or SpO_2_ < 90%) [19], severe hypoxemia (PaO_2_ < 55 mmHg or SpO_2_ < 85%), or severe hypercapnia (PaCO_2_ > 70–80 mmHg) [7]. Our results showed that hypoxemia (SpO_2_ < 92%) and hypercapnia (PetCO_2_ > 60 mm Hg) occurred in approximately one-third of the NI-SV patients, most of whom were treated effectively with transient MV. Due to persistent hypoxemia, only two NI-SV patients (5.7%) were conversed to intraoperative intubation. In another NI-SV patient, the procedure was converted to intubation due to conversion to open thoracotomy. The incidence of conversion to intubation in NI-SV patients was 8.6% in this study, within the reported range of 2–10% [20]. Hypoxemia occurred in 8.6% of the I-MV patients in this study, similiar to the 10–30% reported in the literature [21], and the condition was corrected by repositioning the DLT or double-lung ventilation. Thus, a significant difference was observed in intraoperative airway intervention events between the two ventilation strategies.

The use of the NI-SV as an alternative to the I-MV in VATS aims to reduce postoperative complications and promote rapid recovery, as demonstrated in previous studies [22–24]. Nonetheless, our results from the analysis of the intention-to-treat population showed comparable incidences of intubation-related injuries and PPCs between the two ventilation strategies. The advantage of reducing postoperative complications with the NI-SV strategy was also denied by a recent study [25]. Additionally, the rates of conversion to open thoracotomy were comparable between the NI-SV (2.9%) and I-MV (5.7%) patients, similar to the 4.9% reported in the literature [26].

The NASA-TLX is useful and sensitive for determining the perceived difficulty of a specific surgical technique [27]. Unsafe airways (abnormal oxygenation, conversion to intubation, etc.) and poor surgical conditions (accidental body movement, poor lung collapse, etc.) are the primary concerns when administering the NI-SV strategy for VATS. The comparison of the overall NASA-TLX scores and the dimensional scores from surgeons between the two ventilation strategies indicated that intraoperative airway intervention events and the complication of coughing did not change the difficulty of performing the operation. A similar surgical field exposure score was also reported by surgeons between the two ventilation strategies. The higher scores of the overall NASA-TLX and the dimensional scales from anesthesiologists indicated that they experienced more workload when administering NI-SV anesthesia, especially on the dimensions of temporal demand and effort (*p* ≤ 0.001), which might be related to being vigilant in the surgical process and intraoperative airway intervention events. These findings also support the opinion that the NI-SV technique requires more experience, preparation and vigilance in thoracic surgery [12]. A previous study revealed that a physician’s willingness to approve a treatment plan declined with a NASA-TLX total score ≥ 55 [28]. The 75% overall NASA-TLX score from anesthesiologists when administering the NI-SV was 52 in this study, implying a lower willingness to administer the NI-SV in VATS. This might be a potential factor that limits the use of the NI-SV for VATS in clinical practice.

This study had several limitations. First, the study was performed in a single center with a small sample from the selected population, and by several physicians who had greater willingness and experience in carrying out the NI-SV for VATS. Moreover, the anesthesiologists and surgeons were not blinded during the operation or postoperative care. To some extent, there was selective bias, especially on the NASA-TLX assessment. Second, whether the surgical procedure was performed under NI-SV or I-MV anesthesia was the same. We regard the NI-SV strategy as a challenging technique. This trial was designed mainly to focus on intraoperative airway intervention events rather than enhanced recovery after surgery. Third, a combination of arterial blood gas analysis parameters might be appropriate for oxygenation management during NI-SV anesthesia. For safety purposes, persistent SpO_2_ < 92% and PetCO_2_ > 60 mmHg were considered as indications for conversion to intubation when administering the NI-SV strategy because arterial blood gas analysis was not routinely performed in this study.

## Conclusions

NI-SV anesthesia was feasible for VATS in the selected patients, with a greater incidence of intraoperative airway intervention events than traditional I-MV anesthesia, and with greater effort required for anesthesiologists but not for surgeons during surgery. Further multicenter controlled clinical trials with large sample sizes are warranted to determine the safety and the generalizability of the NI-SV in VATS.

### Electronic supplementary material

Below is the link to the electronic supplementary material.

Supplementary Material 1

## Data Availability

The datasets used and analyzed during the current study are available from the corresponding author on reasonable request.

## Abbreviations

ASA: American Society of Anesthesiologists
BMI: Body mass index
BIS: Bispectral index
CI: confidence interval
CONSORT: Consolidated Standards of Reporting Trials
DLT: Double-lung tube
ICU: intensive care unit
I-MV: Intubaed mechanical ventilation
IPPV: Intermittent positive pressure ventilation
LMA: Laryngeal mask
MV: Mechanical ventilation
NI-SV: Non-intubated spontaneous ventilation
NASA-TLX: National Aeronautics and Space Administration Task Load Index
OR: odds ratio
PACU: post-anesthetic care unit
PCIA: Patient-controlled intravenous analgesia
PetCO_2_: End tidal carbon dioxide pressure
PPCs: Postoperative pulmonary complications
RCT: Randomized controlled trial
RR: Respiratory rate
SpO_2_: Pulse oxygen saturation
SIMV: Synchronized intermittent mandatory ventilation
SV: Spontaneous ventilation
TPVB: Thoracic paravertebral block
TV: Tide volume
VATS: Video-assisted thoracoscopic surgery
