# Supplementary material for: Nonintubated spontaneous ventilation versus intubated mechanical ventilation anesthesia for video-assisted thoracic surgery in terms of perioperative complications and practitioners’ workload assessments: a pilot randomized control study
Source: BMC Anesthesiol. 2024 Mar 12;24:99. doi: 10.1186/s12871-024-02481-1 (PMC10929236; doi:10.1186/s12871-024-02481-1)
Supplement: Supplementary file 1 — Supplementary Material 1 [file 12871_2024_2481_MOESM1_ESM.docx]

| **Supplementary table 1.** Patient surgical and anesthetic characteristics | | | | | | |
| --- | --- | --- | --- | --- | --- | --- |
|  | Intention-to-Treat | |  | Per-Protocol | |  |
|  | NI-SV  (n = 35) | MV  (n = 35) | *p* value | NI-SV  (n=32) | MV  (n=33) | *p* value |
| Surgery type |  |  | 0.602 |  |  | 0.511 |
| Wedge resection | 14(40.0) | 10(28.6) |  | 14(43.8) | 10(30.3) |  |
| Segmentectomy | 5(14.3) | 6(17.1) |  | 4(12.5) | 6(18.2) |  |
| Lobectomy | 16(45.7) | 19(54.3) |  | 14(43.8) | 17(51.5) |  |
| Surgical field exposure score | 1 (1-2) | 1 (1-2) | 0.579 | 1 (1-2[) | 1 (1-2) | 0.336 |
| Duration of surgery (min) | 77(64-88) | 77(71-85.5) | 0.672 | 74(63-86.5) | 77(70-84) | 0.462 |
| Duration of anesthesia (min) | 108(98-119) | 110(101.5-118) | 0.689 | 105.5(97-116) | 108100-115() | 0.503 |
| Propofol requirements (mg) | 409(345-459.5) | 405(366-517) | 0.459 | 404.5(335.5-432) | 405(365-507) | 0.279 |
| Propofol for induction | 130(120-145) | 120(120-140) | 0.601 | 130(120-145) | 120(120-140) | 0.428 |
| Propofol for maintenance | 280(212.5-331) | 285(245-383.5) | 0.229 | 261(204-323) | 263(245-356) | 0.109 |
| Sufentanil requirements (μg) | 25(23-29) | 33(30-35) | < 0.001 | 25(23-28) | 33(30-35) | < 0.001 |
| Sufentanil for induction | 10(10-15) | 20(20-20) | < 0.001 | 10(10-15) | 20(20-20) | < 0.001 |
| Sufentanil for maintenance | 13(11-17) | 13(10-15) | 0.296 | 13(10.5-16) | 13(10-15) | 0.579 |
| Remifentanil requirements(μg) | 299(275-337) | 327(279-377) | 0.242 | 291.5(273.5-337) | 327.6(278-365) | 0.315 |
| Minimum intraoperatove SpO_2_ (%) | 95.5(91.5-96.7) | 95.4(96.4-97.4) | 0.013 | 95.5(92-96) | 96(95-97.5) | 0.021 |
| Maximum intraoperatove PetCO_2_ (mmHg) | 56(53-60.5) | 38(35-41) | **<** 0.001 | 56(53.5-60.5) | 38(35-41) | < 0.001 |
| Extubation time (min) | 9(8-12) | 17(14-19) | **<** 0.001 | 8.5(7.5-11) | 16(14-19) | < 0.001 |
| PACU stay (min) | 23(19-27) | 27(23-32) | 0.035 | 22(18-26) | 27(23-31) | 0.014 |
| Postoperative ICU admission | 0(0) | 0(0) | 1.000 | 0(0) | 0(0) | 1.000 |
| Data are presented median (interquartile range) or number (percentage) when appropriate.  SpO_2_: oxygen saturation; PetCO_2_: end-tidal carbon dioxide pressure; PACU: postanesthetic care unit; ICU: intensive care unit.. | | | | | | |
